# Antioxidant and Cytotoxic Activity of a New Ferruginan A from *Olea ferruginea*: *In Vitro* and *In Silico* Studies

**DOI:** 10.1155/2022/8519250

**Published:** 2022-01-20

**Authors:** Zafar Ali Shah, Adil A. H. Mujawah, Irfan Ullah, Abdur Rauf, Umer Rashid, Anees Ahmed Khalil, Syed Muhammad Mukarram Shah, Aini Pervaiz, Farzana Shaheen, Yahya S. Al-Awthan, Muhammad Nasimullah Qureshi, Mohammed A. Al-Duais, Omar Bahattab, Zainab M. Almarhoon, Yahia N. Mabkhot, Mohammad S. Mubarak

**Affiliations:** ^1^Department of Chemistry, University of Swabi, Swabi, 23561 Khyber Pakhtunkhwa, Pakistan; ^2^Department of Chemistry, College of Science and Arts, Qassim University, Ar Rass 51921, Saudi Arabia; ^3^Department of Chemistry, COMSATS University Islamabad, Abbottabad Campus, 22060 Abbottabad, Pakistan; ^4^University Institute of Diet and Nutritional Sciences, Faculty of Allied Health Sciences, The University of Lahore, Pakistan; ^5^Department of Pharmacy, University of Swabi, Swabi, 23561 Khyber Pakhtunkhwa, Pakistan; ^6^H.E.J. Research Institute of Chemistry, International Center for Chemical and Biological Sciences, University of Karachi, Kararchi 75270, Pakistan; ^7^Department of Biology, Faculty of Science, University of Tabuk, Tabuk, Saudi Arabia; ^8^Department of Biology, Faculty of Science, Ibb University, Ibb, Yemen; ^9^Department of Biochemistry, Faculty of Science, University of Tabuk, Tabuk, Saudi Arabia; ^10^Biochemistry Unit, Chemistry Department, Faculty of Science, Ibb University, Ibb, Yemen; ^11^Department of Chemistry, College of Science, King Saud University, P.O. Box 2455, Riyadh 11451, Saudi Arabia; ^12^Department of Pharmaceutical Chemistry, College of Pharmacy, King Khalid University, Abha, Saudi Arabia; ^13^Department of Chemistry, The University of Jordan, Amman 11942, Jordan

## Abstract

Studies of the ethyl acetate extract bark extract of *Olea ferruginea* led to the isolation of one new compound Ferruginan A (1) in addition to two known compounds, Ferruginan (2) and cycloolivil (3). Structures of the isolated compounds were confirmed by mass spectrometry (MS) and NMR spectral data. The ethyl acetate fraction and compounds (1–3) were evaluated against breast cancer cell line (MCF-7) and as antioxidants using the free radical scavenging assay. Results revealed that compound 2 exhibits significant antioxidant activity with an IC_50_ value of 21.74 *μ*g/mL. In addition, the ethyl acetate fraction showed good cytotoxic activity (79.31% inhibition at 250 *μ*g/mL), whereas compounds 1–3 exerted mild cytotoxic activity (IC_50_ = 8.03–12.01 *μ*g/mL) as compared to the standard (IC_50_ = 4.41 *μ*g/mL) against MCF-7. Docking studies suggested that antioxidant activity is due to the chelation of compounds with copper present in the active site of tyrosinase. These results suggest that the extract exhibits considerable antioxidant activity, and the isolated compounds exert moderate anticancer activity.

## 1. Introduction

Natural products obtained from terrestrial and marine plants have been used as a source of traditional medicines since ancient times. According to the World Health Organization (WHO), people of developing countries rely on natural products for their health care. In the last century, natural products such as Taxol, docetaxel, morphine, cyclosporin A, quinine, simvastain, and lovastatin, among others, have been successfully marketed as safe drugs for the treatment of various diseases. In this respect, around 40% of recent drugs have been developed from natural products [1]. Phenolic compounds, commonly present in fruits and vegetables, are highly bioactive. Currently, these compounds are gaining much attention due to their broad application in the health care systems of several countries. These compounds significantly cure diseases related to free radical-induced stress, cancer, renal, and cardiovascular diseases and act as anti-inflammatory and hepatoprotective [2]. Due to their outstanding benefits, there is an increasing demand for these compounds in the pharmaceutical industry. Additionally, the scientific community is paying much attention to natural products [3].

*Olea ferruginea* is a 10- to 50-feet tall tree belonging to the genus *Olea* and family Oleaceae. This evergreen plant grows in the mountainous areas of Afghanistan, Kashmir, and Pakistan [4]. In Pakistan, the plant is predominantly found in the hilly regions of Azad Kashmir, Khyber Pakhtunkhwa, and Rawalpindi [5]. Traditionally, the stem bark of *Olea ferruginea* has been prescribed for the treatment of fever [6], whereas fresh leaf extracts of this plant were used by people suffering from bleeding gums, toothache, skin diseases, skeletal muscular, and dental problems [7, 8]. In addition, the fruit of *O. ferruginea* is a rich source of oil, which can be applied as massage ointment to relieve pain during dislocated bones and rheumatism [9]. Furthermore, research findings indicated that various parts of this plant have shown significant antimalarial, anti-inflammatory, antiperiodic, antileprosy, astringent, antidiabetic, and antiasthmatic properties [10].

The genus *Olea* is represented by nearly 35 species, and *Olea europaea* L. is the most cultivated around the globe for its oil production [11]. Within this context, extracts of *Olea* plant contain numerous phenolic compounds, flavonoids and lignin, and secoiridoid. Due to the significant phenolic content (17–23%), this plant exhibits antioxidant, anticancer, and antibacterial properties [12, 13]. Biophenols isolated from other *Olea* species reduce cancer growth by various mechanisms. These compounds can either cause a cell cycle arrest in a specific phase or inhibit signaling pathways, which promote proliferation of cells [14]. Furthermore, the increasing demand of olive oil encouraged cultivation of *Olea europaea* in several regions of the world and/or finding alternative sources of olive oil production [15]. For this purpose, *Olea ferruginea*, also known as wild-type olives, has been investigated as a potent source of ample amounts of olive oil. Olive oil production from wild-type olives will not only help in maintaining balance between demand and supply but also improve the national economy and human health [16]. In this respect, studies are required to authenticate the health-promoting benefits and oil quality and production of *Olea ferruginea* (wild olive) [17]. Although numerous studies have dealt with the local cultivars, less attention has been given to the phytochemistry and health-promoting benefits of the plant [15, 18]. Recently, we have reported on the ethyl acetate extract and the isolation of a new compound, Ferruginan, having antibacterial and antileishmanial activity [19]. Based on the preceding discussion, and in view of the importance of *Olea ferruginea* and lack of studies pertaining to its health benefits, the current study was designed to evaluate the antioxidant and anticancer activity of ethyl acetate extract of *O. ferruginea* and to search for new natural compounds present in it having potent antioxidant and chemopreventive properties.

## 2. Material and Methods

### 2.1. General Experimental Procedures

UV-Vis and IR records of isolated compounds were obtained using a spectrophotometer and a Bruker FT-IR spectrophotometer (Japan). Mass spectral data were acquired by employing a FAB-MS spectrometer (Thermo Scientific, United State). Nuclear magnetic resonance spectroscopic data were obtained with the aid of a Bruker Avance (Germany): ^1^H (400 MHz) and ^13^C NMR (100 MHz). Isolated compounds were purified using polar silica gel purchased from Merck-Germany (mesh size of 70–230), and purity was checked by precoated TLC and visualized under UV light.

### 2.2. Plant Material

The *O. ferruginea* plant was collected from the Agriculture Research Institute Tarnab, Peshawar division. Steam, twigs, and leaves were separated and dried in a dark room.

### 2.3. Extraction and Isolation

Crude extract and various fractions were obtained according to our previously reported method [20]. For the isolation of compounds 1–3, 200 g of ethyl acetate extract was loaded on a silica gel column 60 (0.0062-0.200 mm; Merck), using a mixture of *n*-hexane : EtoAC (100 : 0-0 : 100) as a mobile phase. Compound 1 was obtained using *n*-hexane : EtoAC (70 : 30), while compounds 2 and 3 were obtained using *n*-hexane: ethyl acetate(80 : 20-30 : 70).

#### 2.3.1. Ferruginan A (1)

Compound 1 was isolated as a white amorphous powder (80.23 mg): yield = 15%; mp = 124°C; [*α*]_D_^25^ = +210 (*c* = 2.0, CH_3_OH); IR (KBr) = 1027 cm^–1^ for C = O stretching, 1260 cm^–1^, 1462 cm^–1^ for aromatic C = C stretching, and 2900 cm^–1^ for C − H stretching; UV (*λ*_max_) = 283 nm, 230 nm; ^1^H and ^13^CNMR data (see Table 1); and FAB − MS = *m*/*z* 417.1 [M + H]^+^.

### 2.4. Bioactivity

The antioxidant activity of ethyl acetate fractions and compounds 1–3 was determined using the 2,2-diphenyl-1-picrylhydrazyl (DPPH) radical scavenging assay [21]. According to this assay, 7% of DPPH stock solution having a violet color was prepared in methanol. A mixture of 7% DPPH solution (1 mL), extracts and compounds with a concentration of 15–30 *μ*g/mL, and ascorbic acid (positive control) was placed in a labeled test tube for 30 min at room temperature. The optical density (*λ*_max_ = 517 nm) was measured against a standard by means of a UV/visible spectrophotometer; experiments were carried out in triplicate. The relative radical scavenging activity (%) was calculated as [1–absorbance of solution with sample and DPPH/absorbance of solution with DPPH] × 100.

The cytotoxicity study of ethyl acetate extract and compounds 1–3 against MCF-7 was carried out by using the (3-(4,5-dimethylthiazol-2-yl)-2,5-diphenyltetrazolium bromide) MTT assay according to a procedure described by Shah et al. [20]. Reduction of the salt to formazan insoluble crystals is the conformation of inhibitory effect of the extract/compounds. Briefly, the MCF-7 cell line at a concentration of 3 × 10^4^ was seeded in a 96-well plate and incubated at 37°C overnight under 5% CO_2_. On the next day, the original medium was substituted with fresh medium containing 15–30 *μ*g/mL of test samples and subjected to incubation under similar conditions for 48 h. After 48 h incubation, the MTT and fresh medium was added to achieve 0.5 mg/mL, and the plates were further incubated for 4 h. The formazan crystals were dissolved in DMSO, and absorbance was measured at 550 nm using a spectrophotometer (Thermo Fisher Scientific). The percent MCF-7 cell inhibition was calculated using the following formula:

(1) $$\begin{array}{c} \text{Percent inhibition}=\left[\left(\text{OD of the treated well}–\text{OD of untreated well}\right)/\text{OD of the untreated well}\right]\times 100. \end{array}$$

### 2.5. Docking Studies

For docking simulations, we employed the Molecular Operating Environment-2016 (MOE) software. Three-dimensional (3-D) structure of tyrosinase in complex with the antioxidant ligand, kojic acid, having accession code No. 5I38 was obtained from the Protein Data Bank. Preparation of downloaded enzymes for binding site determination, 3-D protonation, and energy minimization was conducted as per previously reported methods [22, 23]. Structures of the compounds were built using the builder option in the MOE software. The built structures were then energy minimized using the MMFF94X force field in addition to 0.0001 gradient and database. The docking study was carried out using validated parameters including placement/refinement stage and scoring/rescoring functions. Interpretation of docking results was achieved using MOE and discovery studio visualizer.

## 3. Results and Discussion

### 3.1. ^1^H and ^13^C NMR

**Figure 1:**
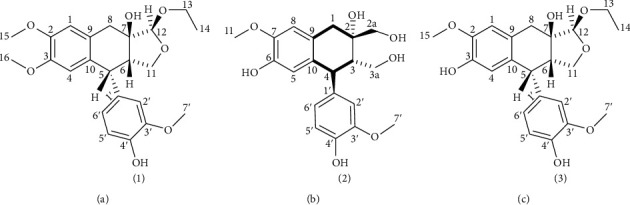
Structures of compounds 1–3.

Bioassay-guided fractionation of *O. ferruginea* crude extract and isolation of compounds from the most active fraction, ethyl acetate fraction, resulted in the separation of Ferruginan A (1), a new lignin, having [M + H]^+^ peak at *m*/*z* 417.1 in the FAB-MS spectrum. The ^1^H-NMR (400 MHz) spectrum showed that compound 1 has five aromatic protons at *δ*_H_ 6.57 (2H, s, H-1, 4), 6.60 (1H, s, H-2′), 6.64 (1H, dd, *J* = 8.2, 1.2 Hz, H-6′), and 6.80 (1H, d, *J* = 8.2 Hz, H-5′) and three methine protons at *δ*_H_ 2.51–2.59 (m, H-6), 3.86 (d, *J* = 12.0 Hz, H-5), and 4.92 (1H, s, H-12). The spectrum also revealed that the compound contains twelve methyl protons at *δ*_H_ 1.17 (3H, t, *J* = 7.2 Hz, H-14), 3.75 (6H, s, H-15, 16), and 3.78 (3H, s, H-7′), along with six methylene protons at *δ*_H_ 2.82 (1H, d, *J* = 16.4 Hz, H-8a), 3.36 (1H, d, *J* = 16.8 Hz, H-8b), 3.72–3.75 (1H, m, H-11a), 3.75–3.78 (1H, m, H-11b), 3.48–3.51(1H, m, H13a), and 3.82–3.85 (1H, m, H-13b). The positions of protons were confirmed by using the heteronuclear single-quantum coherence spectroscopy (HSQC), which showed the aromatic protons at C-4 (*δ*_c_ 110.0), C-1 (*δ*_c_ 112.0), C-2′ (*δ*_c_ 113.2), C-5′ (*δ*_c_ 116.7), and C-6′ (*δ*_c_ 122.3), methine protons at C-5 (*δ*_c_ 45.4), C-6 (*δ*_c_ 49.1), and C-12 (*δ*_c_ 109.5), methylene protons at C-8 (*δ*_c_ 37.0), C-13 (*δ*_c_ 64.0), and C-11 (*δ*_c_ 71.3), and methyl protons at C-14 (*δ*_c_ 15.4), C-15 (*δ*_c_ 56.3), C-16 (*δ*_c_ 56.3), and C-7′ (*δ*_c_ 56.5). The broadband decoupled ^13^C-NMR spectrum of 1 revealed that besides the six aromatic carbons, three methine carbons, six methylene carbons, and four methyl carbons, the compounds contains eight quaternary carbons at *δ*_c_ 79.2 (C-7), 137.0 (C-1′), 130.9 (C-10), 133.1 (C-9), 145.2 (C-3), 145.0 (C-2), 149.2 (C-3′), and 147.3 (C-4′). Based on FAB-MS, and on ^1^H and ^13^C-NMR spectral data, the molecular formula (C_23_H_28_O_7_) for compound 1 was established. The ^1^H- and ^13^C-NMR data revealed one extra methoxy group that appeared as singlet at *δ*_H_ 6.57, and the up-field shifting of C-4 signal, from *δ*_c_ 116 to 110 (Figure 1).

**Figure 2:**
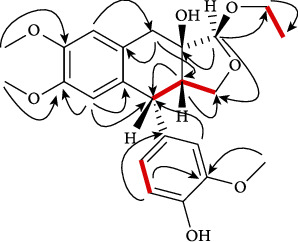
Key 2-D NMR correlations of compound 1.

In the HMBC spectrum, H-12 of tetrahydrofuran showed correlation with the tertiary alcoholic carbon C-7 (*δ*_c_ 79.2) and H-6 of the tetrahydronaphthalene moiety with C-11 (*δ*_c_ 71.3) of tetrahydrofuran, which supported the linkage of tetrahydrofuran ring with tetrahydronaphthalene ring. Similarly, correlations of methoxy protons H-15, 16, and 7′ with C-2 (*δ*_c_ 145.0), C-3 (145.2), and C-3′ (*δ*_c_ 149.1), respectively, confirmed their positions. Connection of the ortho-methoxy phenol with tetrahydronaphthalene moiety was verified by the connection between H-2′ and C-5 (*δ*_c_ 45.4) (Figure 2). The three-spin system was detected in the COSY 45° spectrum (Figure 2). The correlation of H-5, H-6, and H-11 with each other was noted in the spin system I, while the correlation between H-5′ and H-6′ was noticed in the spin system II. The connection between H-13 and H-14 was noticed in the spin system III. ^1^H- and ^13^C-NMR data for 1 are presented in Table 1.

### 3.2. Bioactivity

Ethyl acetate fraction and isolated compounds 1–3 were tested for free radical scavenging activity using the DPPH assay. Results showed that the ethyl acetate fraction and compound 2 exhibit potent antioxidant potential with IC_50_ values of 79.03 and 21.74 *μ*g/mL, respectively, while compounds 1 and 3 exerted good scavenging activity (Table 2). The antioxidant potential data of compound 2 matched those reported [24]. The potent antioxidant activity may be due to the presence of phenolic compounds in ethyl acetate (polar) fraction, and five hydroxyl groups (-OH) in compound 2, which support the concept that compounds having higher number of hydroxyl groups (-OH) or other H-donating groups (-NH; -SH) have display antioxidant activity [21].

The anticancer potential of ethyl acetate fraction and compounds 1–3 against breast cancer cell line (MCF-7) was also investigated. Results listed in Table 2 revealed that the percentage inhibition of MCF-7 cell line increases as the concentration (50–250 *μ*g/mL) of ethyl acetate extract and doxorubicin (std) increase. Results also showed that compounds 1–3 exhibit good potential against MCF-7 cell lines (Table 3). These findings support the notion that natural products of *Olea* species have been shown to exhibit cytotoxic activity toward human cancer cells with little or no effect on normal cells [25].

### 3.3. Docking Studies

**Figure 3:**
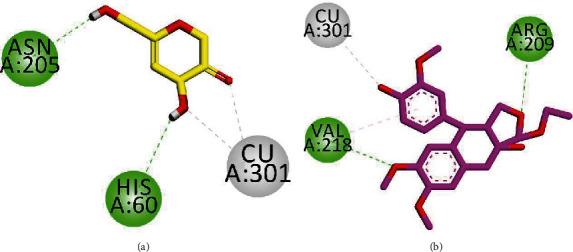
2-D interaction plot of kojic acid ((a) yellow) and isolated compound 1 ((b) pink).

**Figure 4:**
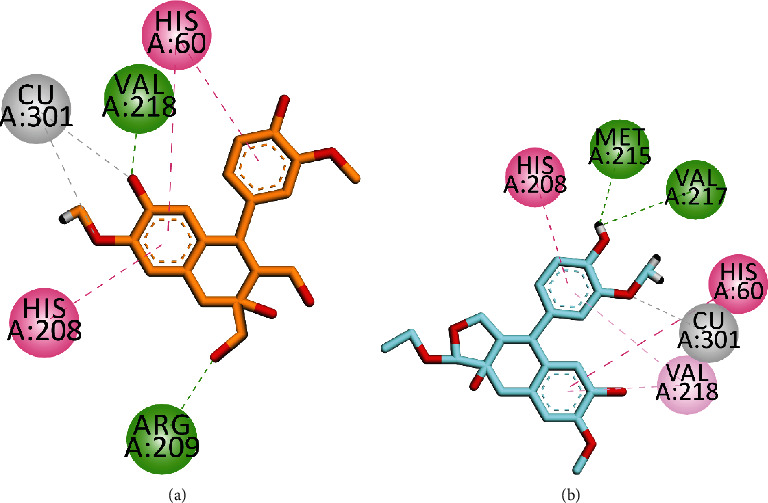
2-D interaction plot of compound 2 ((a) orange) and isolated compound 1 ((b) turquoise).

We performed docking studies on tyrosinase, which is a widely dispersed copper containing enzyme. This enzyme has a fundamental role in catalyzing the hydroxylation of mono- and diphenol oxidation reactions [26]. Three-dimensional (3-D) structure of tyrosinase in complex with the antioxidant ligand kojic acid having accession code No. 5I38 was obtained from the Protein Data Bank. We employed the MOE (Molecular Operating Environment-2016) software for docking simulations. After validation of the docking protocol by redock method (RMSD = 0.85 Å), we docked all three isolated compounds into the binding site of 5I38. Two-dimensional (2-D) interaction plots of the ligand-enzyme complexes are shown in Figures 3 and 4. The native compound, kojic acid, is an inhibitor of tyrosinases from various origins such as bacteria and mushrooms [27]. Additionally, 2-D interaction plots indicate that kojic acid chelates with copper ions in a bidentate manner *via* hydroxyl and carbonyl groups. Moreover, it forms hydrogen bond interactions with Asn205 (active site entrance residue) and His60 (Figure 3(a)). Isolated compound 1 chelates with Cu ions and forms hydrogen bond interactions with Val218 present on a loop adjacent to Cu301 [27]. It also displayed a conventional hydrogen bond interaction with Arg209 positioned near the entrance of the active site (Figure 3(b)).

As for compound 2, the 2-D interaction plot showed that it chelates with copper ions in a bidentate manner *via* hydroxyl and methoxy oxygen atoms. Conventional hydrogen bond interactions were also observed with Arg209 and Val218 *via* hydroxyl groups. His60 and His208 displayed *π* − *π* interactions with phenyl groups (Figure 4(a)). Compound 3 is oriented toward Cu ion *via* the methoxy group and forms hydrogen bond interactions with Met215 and Val217. His60 and His208 displayed *π* − *π* interactions with phenyl groups, while val218 displayed *π*-alkyl interactions (Figure 4(b)).

## 4. Conclusions

In addition, findings from this investigation reveal that the isolated compounds and BEPS exhibit significant anti-inflammatory effects in both *in vitro* and *in vivo* anti-inflammatory models, which justifies the use of this plant in Algerian folk medicine. In the study, we have succeeded in isolating a new compound (1) and two known compounds (2 and 3) from *O. ferruginea*. In addition, findings from this investigation reveal that the ethyl acetate fraction and compound 2 exhibit potent antioxidant activity. Moreover, the ethyl acetate fraction and compounds 1–3 also showed significant anticancer activity against the breast cancer cell line (MCF-7). Docking studies suggest that the isolated compounds show tyrosinase inhibition by chelating with copper present in the active site and thus exhibited antioxidant activity.

## Figures and Tables

**Table 1:** ^1^H and ^13^C NMR data of Ferruginan A (1) (*δ* in ppm) in CD_3_OH.

C. No.	^1^H-*δ* (*J*, Hz)	^13^C (*δ*) observed	^13^C (*δ*) Ferruginan	Multiplicity
1	6.57, s	112.0	113.8	CH
2	—	145.0	145.3	C
3	—	145.2	146.3	C
4	6.57, s	110.0	116.7	CH
5	3.86, d, *J*_3,4_ = 12.0 Hz	45.4	45.4	CH
6	2.51-2.59, m	49.1	49.2	CH
7	—	79.2	79.4	C
8	*H*_a_ = 2.82, d, *J*_a,b_ = 16.4 Hz *H*_b_ = 3.36, d, *J*_a,b_ = 16.8	36.9	36.7	CH_2_
9	—	133.1		C
10	—	130.9		C
11	*H*_a_ = 3.72 − 3.75, m; *H*_b_ = 3.75 − 3.78, m	71.3	71.3	CH_2_
12	4.92, s	109.5	109.5	CH
13	3.48-3.51, m; 3.78-3.85, m	64.0	64.1	CH_2_
14	1.17, t, *J*_13,14_ = 7.2 Hz	15.4	15.5	CH_3_
15	3.75, s	56.3	56.3	CH_3_
16	3.75, s	56.3	—	—
1'	—	137.0	137.0	C
2'	6.60, br s	113.2	113.1	CH
3'	—	149.2	149.2	C
4'	—	147.3	147.3	C
5'	6.80, dd, *J*_5′,6,2^″^_ = 8.2 Hz	116.7	116.7	CH
6'	6.64, d, *J*_5′,6′_ = 8.2, 1.2 Hz	122.3	122.3	CH
7'	3.78, s	56.5	56.5	CH_3_

**Table 2:** *In vitro* antioxidant and cytotoxic activity of ethyl acetate extract and compounds (1–3) of *O. ferruginea.*

Compound No.	Antioxidant activity IC_50_ value (*μ*g/mL)	MCF-7 assay IC_50_ value (*μ*g/mL)
Ethyl acetate extract	79.03	>100
Ferruginan A (1)	28.74	12.01
Cycloolivil (2)	21.74	8.03
Ferruginan (3)	25.74	10.41
Ascorbic acid	22.80	N/A
Doxorubicin	N/A	4.41

N/A: not applicable.

**Table 3:** *In vitro* cytotoxic activity of ethyl acetate extract of *O. ferruginea* against MCF-7 cell lines at different concentrations.

Concentration (*μ*g/mL)	% inhibition on MCF-7 cell line
	Ethyl acetate extract
50 *μ*g/mL	24.30
100 *μ*g/mL	42.34
150 *μ*g/mL	57.02

## Data Availability

The supporting information is available online (see supplementary file).

## Supplementary Materials

Supplementary Materials Spectroscopic data related to isolated compounds 1–3 are available online.
