# Supplementary material for: Antioxidant and Cytotoxic Activity of a New Ferruginan A from Olea ferruginea: In Vitro and In Silico Studies
Source: Oxid Med Cell Longev. 2022 Jan 20;2022:8519250. doi: 10.1155/2022/8519250 (PMC8794655; doi:10.1155/2022/8519250)
Supplement: Supplementary Materials — Spectroscopic data related to isolated compounds 1–3 are available online. [file 8519250.f1.docx]

**Antioxidant and cytotoxic activity of a new Ferruginan A from *Olea ferruginea: in vitro* and in Silico Studies**

Zafar Ali Shah^a^, Adil A.H. Mujawah^b^, Irfan Ullah^a^, Abdur Rauf *^a^, Umer Rashid^c^, Anees Ahmed Khalil^d^, Syed Muhammad Mukaram Shah^e^, Aini Pervaiz^e^, Farzana Shaheen^f^, Yahya S. Al-Awthan^g,h^, Muhammad Nasimullah Qureshi^a^, Mohammed A. Al-Duais^i,j^, Omar Bahattab^g^, Zainab M. Almarhoon^k^, Yahia N. Mabkhot^l,^ Mohammad S. Mubarak^m^

^a^Department of Chemistry, University of Swabi, Swabi-23430, Khyber Pakhtunkhwa, Pakistan.

^b^Department of Chemistry, College of Science and Arts, Qassim University, Ar Rass, 51921, Saudi Arabia

^c^Department of Chemistry, COMSATS University Islamabad, Abbottabad Campus, 22060 Abbottabad, Pakistan

^d^University Institute of Diet and Nutritional Sciences, Faculty of Allied Health Sciences, The University of Lahore, Pakistan

^e^Department of Pharmacy, University of Swabi, Swabi-23430, Khyber Pakhtunkhwa, Pakistan.

^f^H.E.J. Research Institute of Chemistry, International Center for Chemical and Biological Sciences, University of Karachi, Karachi, Pakistan

^g^Department of Biology, Faculty of Science. University of Tabuk, Tabuk, Saudi Arabia

^h^Department of Biology, Faculty of Science, Ibb University, Ibb, Yemen

^i^Department of Biochemistry, Faculty of Science, University of Tabuk, Tabuk, Saudi Arabia

^j^Biochemistry Unit, Chemistry Department, Faculty of Science, Ibb University, Ibb, Yemen

^k^Department of Chemistry, College of Science, King Saud University, P.O. Box 2455, Riyadh 11451, Saudi Arabia

^l^Department of Pharmaceutical Chemistry, College of Pharmacy, King Khalid University, Abha, Saudi Arabia

^m^Department of Chemistry, University of Jordan, Amman 11942, Jordan

.

***Corresponding author:** [abdurrauf@uoswabi.edu.pk](mailto:abdurrauf@uoswabi.edu.pk)

**Spectroscopic study of compounds (1-3)**

| No. | Name of Spectra | Page No. |
| --- | --- | --- |
| 1 | FAB-MS spectra of compound **1** | 2 |
| 2 | HRFAB-MS spectra of compound **1** | 2 |
| 3 | ^1^H-NMR spectra of compound **1** | 3 |
| 4 | ^13^C-NMR spectra of compound **1** | 3 |
| 5 | HSQC spectra of compound **1** | 4 |
| 6 | HMBC spectra of compound **1** | 4 |
| 7 | COSY spectra of compound **1** | 5 |
| 8 | EI-MS spectra of compound **2** | 5 |
| 9 | ^1^H-NMR spectra of compound **2** | 6 |
| 10 | EI-MS spectra of compound **3** | 6 |
| 11 | ^1^H-NMR spectra of compound **3** | 7 |


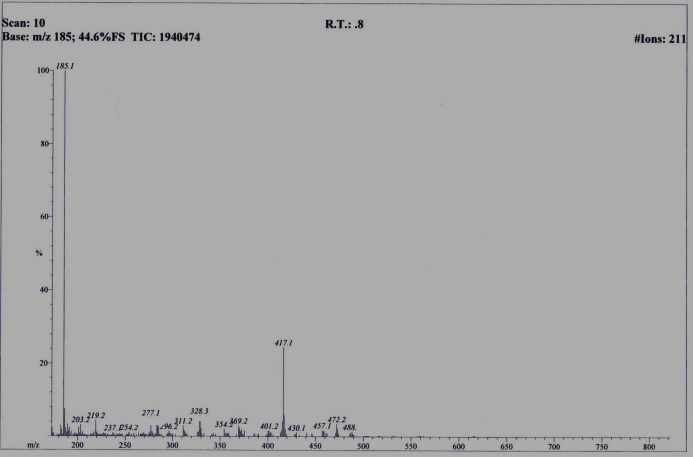


Figure-S1: FAB-MS spectra of compound **1**


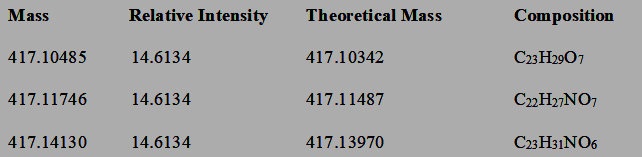


Figure-S2: HRFAB-MS spectra of compound **1**


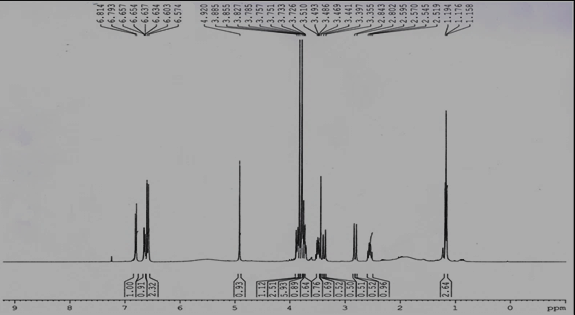


Figure-S3: ^1^H-NMR spectra of compound **1**


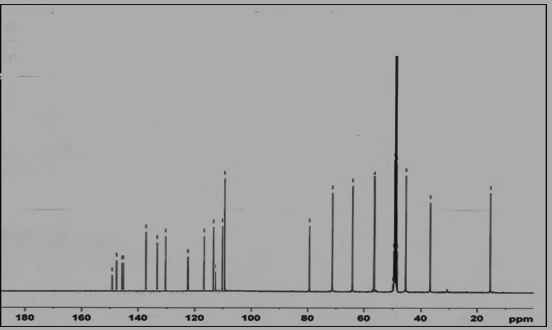


Figure-S4: ^13^C-NMR spectra of compound **1**


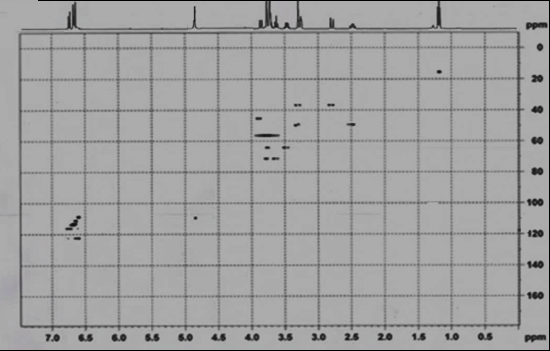


Figure-S5: HSQC spectra of compound **1**


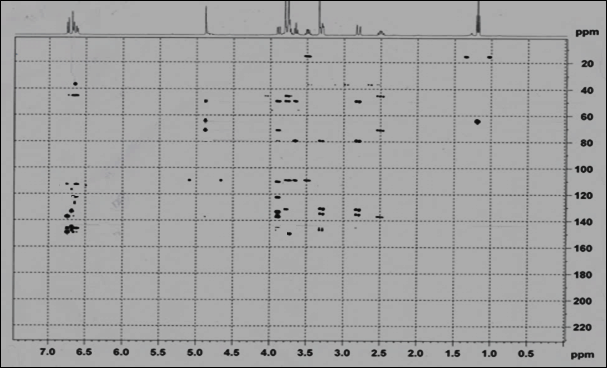


Figure-S6 HMBC spectra of compound **1**


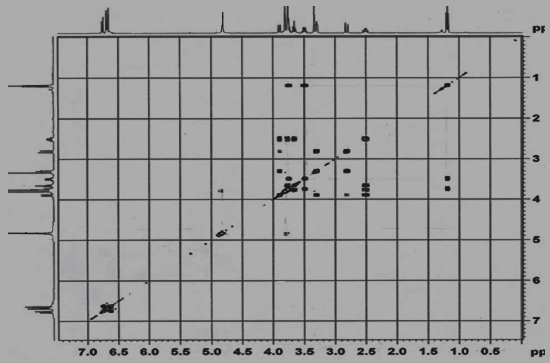


Figure-S7: COSY spectra of compound **1**

Figure-S8: EI-MS spectra of compound **2**


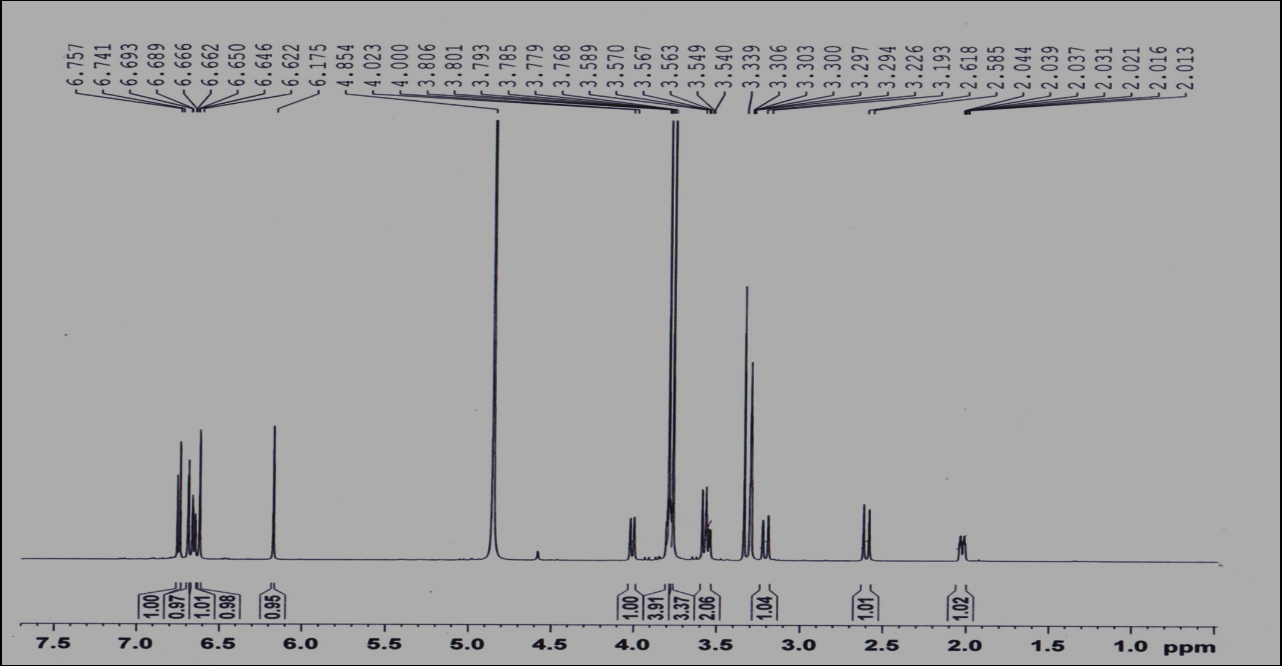


Figure-S9: ^1^H-NMR spectra of compound **2**


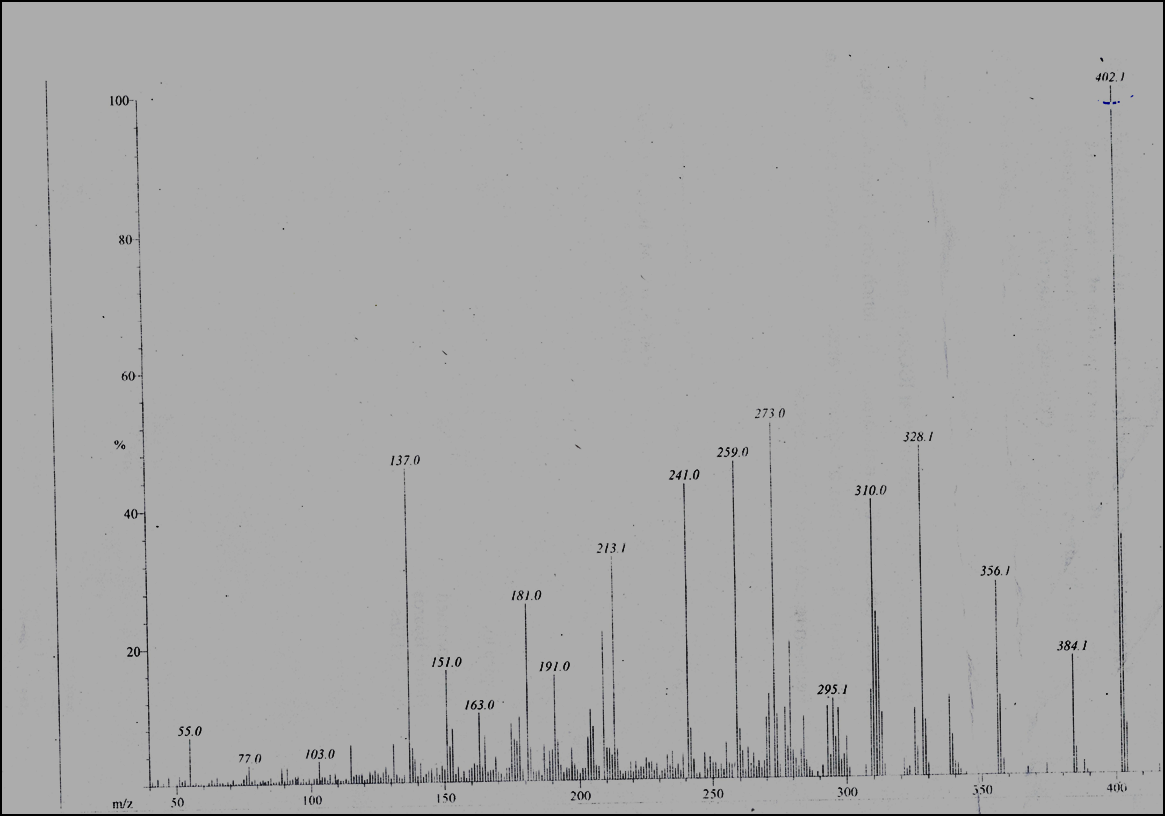


Figure-S10: EI-MS spectra of compound **3**


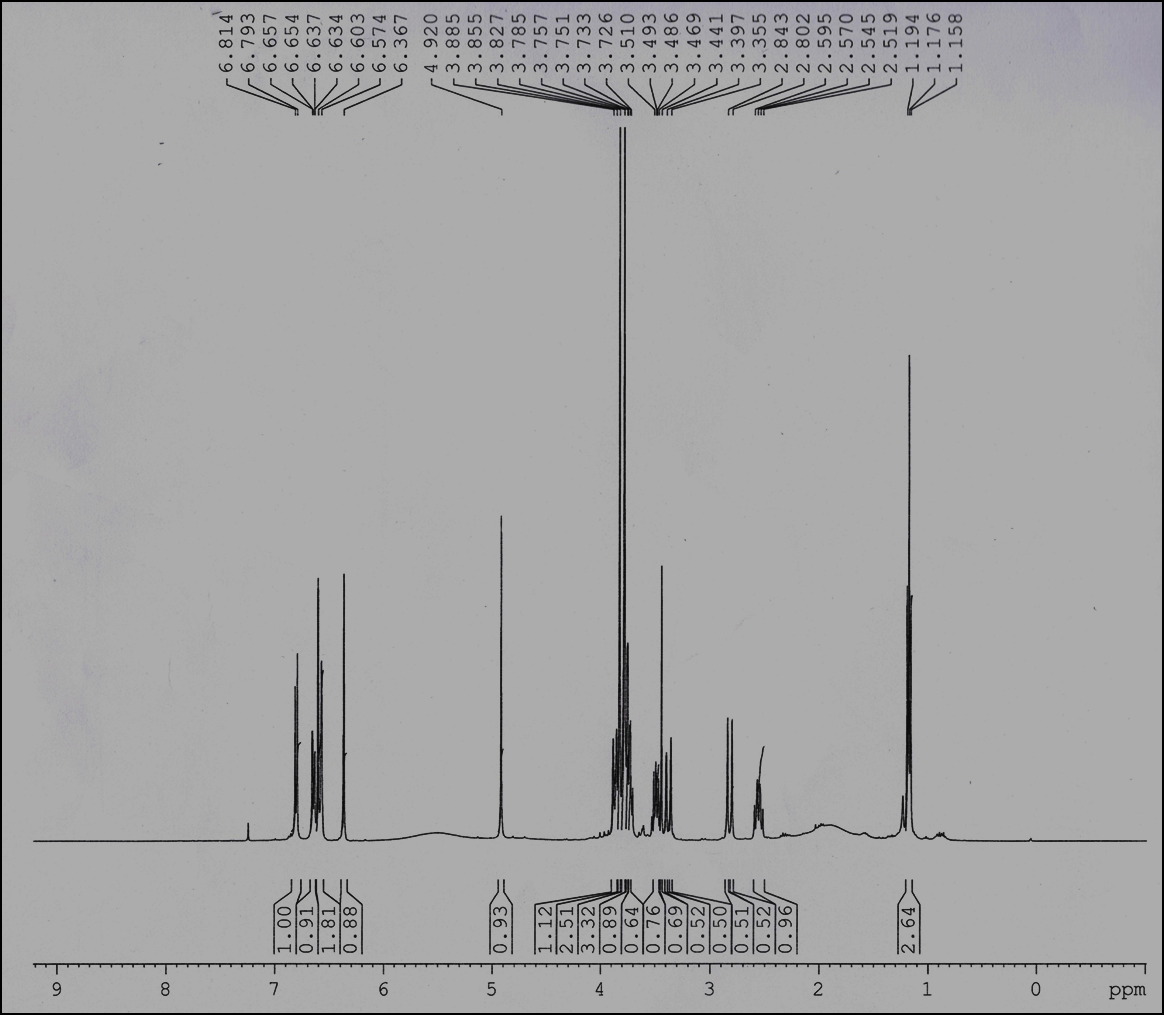


Figure-S11: ^1^H-NMR spectra of compound **3**
